# Human Rhabdomyosarcoma Cell Lines for Rhabdomyosarcoma Research: Utility and Pitfalls

**DOI:** 10.3389/fonc.2013.00183

**Published:** 2013-07-17

**Authors:** Ashley R. P. Hinson, Rosanne Jones, Lisa E. S. Crose, Brian C. Belyea, Frederic G. Barr, Corinne M. Linardic

**Affiliations:** ^1^Department of Pediatrics, Duke University Medical Center, Durham, NC, USA; ^2^Duke University School of Medicine, Durham, NC, USA; ^3^Department of Pediatrics, University of Virginia School of Medicine, Charlottesville, VA, USA; ^4^Laboratory of Pathology, National Cancer Institute, Bethesda, MD, USA; ^5^Department of Pharmacology and Cancer Biology, Duke University Medical Center, Durham, NC, USA

**Keywords:** alveolar, embryonal, human cell line, rhabdomyosarcoma, xenograft

## Abstract

Rhabdomyosarcoma (RMS) is the most common soft tissue sarcoma of childhood and adolescence. Despite intergroup clinical trials conducted in Europe and North America, outcomes for high risk patients with this disease have not significantly improved in the last several decades, and survival of metastatic or relapsed disease remains extremely poor. Accrual into new clinical trials is slow and difficult, so *in vitro* cell-line research and *in vivo* xenograft models present an attractive alternative for preclinical research for this cancer type. Currently, 30 commonly used human RMS cell lines exist, with differing origins, karyotypes, histologies, and methods of validation. Selecting an appropriate cell line for RMS research has important implications for outcomes. There are also potential pitfalls in using certain cell lines including contamination with murine stromal cells, cross-contamination between cell lines, discordance between the cell line and its associated original tumor, imposter cell lines, and nomenclature errors that result in the circulation of two or more presumed unique cell lines that are actually from the same origin. These pitfalls can be avoided by testing for species-specific isoenzymes, microarray analysis, assays for subtype-specific fusion products, and short tandem repeat analysis.

## Introduction

Rhabdomyosarcoma (RMS) is a malignancy that arises from skeletal muscle precursors (1). It is the most common type of soft tissue sarcoma in children and adolescents less than 20 years old, with an incidence of 4.5 cases per million children/adolescents per year (2). There are two major subtypes of RMS, embryonal and alveolar, which differ markedly in their outcomes. Embryonal RMS usually presents in children less than 10 years old and has a 5-year survival of close to 75%. Alveolar RMS, on the other hand, occurs at about the same incidence throughout childhood and adolescence and is associated with a poorer prognosis with a 5-year survival rate of less than 50%. Patients with refractory or relapsed RMS have an even worse prognosis, with survival ranging from 10 to 30% (3).

Despite numerous clinical trials, outcomes for high risk RMS have not improved significantly in the last 30 years. Given the relatively small population of children diagnosed with this disease annually, and the number and timing of new clinical trials, testing new potential treatment agents is limited. *In vitro* cell-line research and *in vivo* xenograft models aid in the basic and preclinical effort to identify potential new treatments. If shown promising, these agents can then be taken into human clinical trials, and compared to standard of care agents. The Pediatric Preclinical Testing Panel (PPTP) is an initiative formed by the National Cancer Institute, working to further characterize and validate available cell lines in multiple kinds of pediatric cancer, including RMS so that preclinical evaluations of new chemotherapeutic agents can be tested (4). Currently, there are 18 embryonal and 12 distinct alveolar human RMS cell lines described in the literature that have been used in more than one study by more than one research group. They differ in their origins, histologies, karyotypes, and methods of validation. They are described below and summarized in Table 1. There are also 16 human RMS cell lines that have been described and used by single research groups (5–17); these are listed in Table 2. [Of note, during revisions of this article an independent list of human and murine RMS cell lines was published (18).] The current article aims to summarize the published RMS cell lines, aid scientists in deciding which lines may be applicable to their research projects, and highlight important historical information and limitations for specific cell lines.

**Table 1:** **Human RMS cell lines reported and used by multiple research groups**.

Cell lines	Origin	Previous treatment	Karyotype/gene fusion status	STR
**EMBRYONAL HISTOLOGY**				
CCA	“Vesical” recurrence	Unknown	Multiple chromosomal rearrangements, Q61L mutation of *KRAS*	Unknown
CT-TC	Primary tumor	Unknown	Unknown	Unknown
HX170c	Paratesticular tumor of 5-year-old male at recurrence	Yes	Near diploid	Unknown
JR-1	Lung metastasis 7-year-old female	Yes	Near tetraploid, *TP53* mutation	Unknown
KF-RMS-1	Bone marrow metastasis	Unknown	Unknown	Unknown
RD	Pelvic mass 7-year-old female	Yes	51-hyperdiploid, *MYC* amplification, Q61H mutation of *NRAS*, *TP53* mutation	Yes
RH6	Lymph node metastasis 16-year-old male	Unknown	Unknown	Unknown
RH12	Buttock tumor	No	Multiple chromosomal rearrangements	Unknown
RH14	Inguinal tumor at recurrence	Yes	Unknown	Unknown
RH36 (Birch)	Paratesticular relapse 15-year-old male	Yes	Unknown	Unknown
RMS559/R31	Unknown	Unknown	V550L mutation of FGFR4	Unknown
RMS-YM	Abdominal mass 2-year-old male	Unknown	Multiple chromosomal rearrangements, upregulation of *MDM2*, *FGFR1*	Unknown
RUCH2	Botryoid vaginal mass 15-month-old female	No	Multiple chromosomal rearrangements	Unknown
RUCH3	Unknown	Unknown	Unknown	Unknown
SCMC-RM2	Bone marrow metastasis of 11-year-old female	Yes	*NMYC* amplification	
SMS-CTR	Pelvic mass 1-year-old	No	Hypertriploid, t(1;13)(q21;q14) and t(2;13)(p25;q14)*	Yes
TTC442	Unknown	Unknown	Unknown	Unknown
TTC516	Unknown	Unknown	Unknown	Unknown
**ALVEOLAR HISTOLOGY**				
CW9019	Unknown	Unknown	t(1;13)	Unknown
D-RHA1	Unknown	Unknown	Unknown	Unknown
KFR	Bone marrow metastasis	No	Pseudodiploid, t(2;13)(q35;q14)^$^	Unknown
RH5	Unknown	No	Unknown	Unknown
RH10	Perineal relapse 5-year-old female	Yes	Hyperdiploid, t(2;13)	Unknown
RH18 fusion-negative	Perineal mass 2-year-old	No	Amplification of 12q13–15 region including MDM2	Yes
RH18 fusion-positive	Perineal mass 2-year-old	No	t(2;13)	Yes
RH28 (RH3)	Axillary metastasis 17-year-old male	No	Near tetraploid t(2;13)	Yes
RH30 (RMS13)	Bone marrow metastasis 16-year-old male	No	t(2;13), *TP53* mutation, amplification of 12q13–15 region including CDK4	Yes
RH41 (RH4)	Lung metastasis 7-year-old female	Yes	t(2;13), *TP53* mutation	Yes
RH65	Relapse 18-year-old female	Yes	Unknown	Unknown
RMZ-RC2	Bone marrow metastasis 2-year-old	Yes	PAX7-FOXO1, *NMYC* amplification	Unknown
TC212	Bone marrow metastasis 16-year-old male	Yes	t(2;13)(q35;q14)^$^	Unknown

*These are not the characteristic translocations associated with ARMS. See text.

*^$^There was a time that the 2q breakpoint was assigned to q35 or q37; this has now been resolved as a q35 breakpoint. See text*.

**Table 2:** **Additional human RMS cell lines reported and used by a single research group**.

Cell lines	Origin	Previous treatment	Karyotype	Additional information	Reference
**EMBRYONAL HISTOLOGY**					
DI-OH1	Primary tumor, pediatric male	Unknown	50, XY, +2, +8, +11	Undetectable telomerase activity	Kleideiter et al. (8)
VK	Primary tumor of 20-year-old female with localized disease	None	46, XX, t(1;11)(p36.31;p11.2), t(10;11)(q22.3;p11.2), t(1;10)(p36.31;q22.3), t(2;17)(q23;p13.3), t(4;19)(q35.1;q13.1), t(14;17)(q32.3;q21.31)		Urumov and Manolova (11)
RMS-GR	Relapsed urogenital tract tumor of 75-year-old male	Yes	59, XY, −1, +der(1)t(1;?)(q31;?), +del(1)(p13), +del(3)(p13), +del(3)(p13), der(3)t(3;19)(p11;q11), +der(8)t(8;?)(p23;?), +M	Upregulation of *mdr1*	Fernandez et al. (6)
TS-RM-1	Unknown	Unknown	Chromosome number 88−98 der(3)t(1;3)(q12;p12–14), 16q-, 17q+, and 21q+		Kaneko et al. (13)
YN	15-year-old male with paratesticular tumor	Unknown	Unknown		Motoyama et al. (12)
**ALVEOLAR HISTOLOGY**					
CB-NJR	Unknown	Unknown	Unknown		Ozkaynak et al. (10)
FL-OH1	Primary tumor, pediatric male	Unknown	60–89, XY, many clonal and non-clonal aberrations, negative for t(1;13), t(2;13)	High telomerase activity	Kleideiter et al. (8)
HA-OH1	Relapsed tumor, pediatric female	Unknown	78–87, XXX, der(1), t(1;13), der(1), t(1;13), i(q), del(3), 4q+, del(6), −13, [cp4]	Low telomerase activity	Kleideiter et al. (8)
HUMEMS	13-year-old female with breast primary, cell line established from pleural fluid		t(2;13)		Ohi et al. (14)
NRS-1	7-year-old female with forearm primary	Unknown	t(2;13)		Ogose et al. (15)
RH7	Primary tumor	Unknown			Morton and Potter (23)
UISO-RS-3	28-year-old female with buttock primary, cell line established from a malignant pleural effusion	Yes	43–49, X, Philadelphia 22 chromosome (70% of metaphase spreads)		Madsen et al. (10)
Not named	14-year-old female with chest wall primary, cell line established from malignant effusion	Unknown	41–49, t(2,13)(q37;q14), double minute chromosomes		Garvin et al. (7)
**PLEOMORPHIC HISTOLOGY**					
HS-RMS-1	26-year-old male with gluteal tumor	Unknown	Pseudotetraploid		Sonobe et al. (16)
HS-RMS-2	85-year-old female with gluteal tumor	Unknown	8 amplified regions including oncogenes JUN, MYC, CCND1, INT2, MDM2, and MALT		Takaoka et al. (17)
**UNKNOWN HISTOLOGY**					
HUS-2	80-year-old female with chest wall mass	No	Modal chromosome number of 58		Cook et al. (5)

## Embryonal RMS Cell Lines

### CCA

CCA was derived from the biopsy of a “vesical” recurrence of embryonal RMS in an 8-year-old Caucasian male (19). Multiple chromosomal rearrangements were identified upon karyotype analysis, with additional defects on chromosomes 1, 4, 6, 8, 9, 10, 11, 12, and 13 (20). CCA cells express vimentin and desmin. These cells can be used to generate xenografts in nude mice subcutaneously or intramuscularly, and form lung metastases when injected intravenously after pretreatment of the mice with cyclophosphamide. CCA cells harbor a Q61L mutation in *KRAS* (21). CCA has been grown in modified Dulbecco’s medium (DMEM) with 10% fetal bovine serum (FBS) (22). As with cell culture in general, it is up to the investigator whether prophylactic antibiotics penicillin and streptomycin are to be included during routine culture.

### CT-TC

This cell line was derived from a primary tumor with an embryonal histology and expresses MyoD, myogenin, and desmin (at very low levels). It was originally developed by Dr. Hajime Hosoi and can be grown in DMEM with 10% FBS (23).

### HX170c

HX170c was established from a paratesticular tumor of a 5-year-old Caucasian male. The patient had been previously treated with vincristine, adriamycin, cyclophosphamide, and radiotherapy. The tumor specimen was designated RMS based on the presence of desmin intermediate filaments, and assigned embryonal histology. HX170c was established simultaneously as a cell line in culture and xenograft directly from the biopsy of a local recurrence 2 months prior to the patient’s death. At early passages, HX170c was cultured *in vitro* on a lethally irradiated layer of mouse fibroblast 3T3 cells; the cell line was later tested and found to contain only human cells. While the tumor biopsy was positive for desmin staining, the HX170c cell line was almost completely negative for this marker when cultured *in vitro*, except when grown to almost complete confluence. Interestingly, HX170c subcutaneous mouse xenografts regained desmin positivity and also appeared histologically similar to the initial tumor biopsy. HX170c cells are near diploid (50 ± 6) (24).

### JR-1

JR-1 was derived from a lung metastasis of a 7-year-old female with a primary uterine mass, previously treated with vincristine, cyclophosphamide, dactinomycin, and radiation therapy (25). It has a poorly differentiated embryonal histology, based on histological appearance of the biopsy specimen. This cell line was grown in RPMI-1640 medium supplemented with FBS. Chromosome analysis revealed a near tetraploid number of chromosomes, ranging from 44 to100 per cell, with 80% showing chromosomal alterations, the most common of which was 13q−. JR-1 cells have been shown to have mutation of the *TP53* gene (26). Antibody staining showed similarities between the cell line and the original tumor, as both stained positive for desmin, vimentin, glycolipid, ganglioside Gq, thy-1 and Gp44; and negative for GFAP, cytokeratin, neurofilament RT97, and myoglobin.

### KF-RMS-1

KF-RMS-1 has an embryonal histology (based on the histologic appearance of its tumor source) and is derived from a bone marrow metastasis. It is grown in DMEM with 10% FBS (22).

### RD

RD was derived directly from biopsy specimens of a 7-year-old female with a pelvic RMS previously treated with cyclophosphamide and radiation and found to have refractory disease (27). It has an embryonal histology based on histologic appearance of the tumor biopsies and the cultured cells, and 51-hyperdiploid chromosomes. RD has been shown to have amplification of the *MYC* oncogene (28), Q61H mutation of *NRAS* (29), and homozygous mutation of *TP53* (30, 31). Houghton et al. studied tumor size following injections of various chemotherapy agents and found that RD demonstrated growth inhibition to vincristine and cyclophosphamide, but no other agents tested (32). RD was also used to test tolfenamic acid, and showed decreased tumor size, decreased cell migration, and decreased expression of Sp specificity transcription factors after treatment (33). As mentioned in the Section “Discussion,” the cell line TE671, which was originally thought to be a medulloblastoma line, was later shown through cytogenetic analysis and DNA fingerprinting to likely be a subclone of RD cells (34). RD cells are one of the most commonly used cell lines in RMS research, can be obtained from ATCC, and are grown in Eagle’s medium with 10% FBS (35).

### RH6

The RH6 cell line was established in the laboratory of Dr. Edwin Douglass at St. Jude’s Children’s Research Hospital (SJCRH) in Memphis, TN directly from a 16-year-old male with an infra-renal lymph node (personal communication, Susan Ragsdale, SJCRH). Of note, it has no relation to the Rh6 xenograft listed in (36). When evaluated using cDNA microarray analysis of the cell line versus original tumor, the RH6 cell line did not cluster within the RMS family of tumors and therefore, is not optimal for testing of this cancer type (37). More recently, other studies do mention that this cell line expresses myogenic markers (38).

Of historical and nomenclature import, all of the “RH” cell lines (sometimes denoted “SJRH”) were generated at SJCRH and can be obtained from a representative of that institution, or from Dr. Peter Houghton at Nationwide Children’s Hospital, Columbus, OH, USA who has maintained these lines. However, during the years in which human RMS cell lines were first being established, two separate laboratories were deriving and naming the cell lines independently. Cell lines established in the laboratory of Dr. Peter Houghton were generated by directly transplanting tumor into mice to establish xenografts, and originally labeled “Rh.” Cell lines established in the laboratory of Dr. Edwin Douglass (and subsequently Dr. David Shapiro) were generated directly from patient samples and labeled “RH.” The upper or lower case designations for these cell line names have since been interchanged in the literature and are no longer preserved.

### RH12

RH12 was derived from a xenograft created from a buttock tumor in a previously untreated patient. The original tumor and corresponding xenograft have an embryonal histology based on their spindled appearances (36). Chromosome analysis showed the addition of multiple chromosomes including 2, 3, 7, 8, 9, 10, 11, 12, 13, 18, 21, and dup (1)(q11q31), +del(2)(q23) (39). In a study whereby tumor growth was measured against various chemotherapy agents, RH12 showed volume regression with vincristine, DTIC, cyclophosphamide, doxorubicin, cis-DDP, and busulfan. No growth inhibition was seen with the use of dactinomycin, mitomycin-C, or bleomycin (32). RH12 xenografts were also used to test the effect of vincristine plus topotecan on tumor growth; the combination showed improvement over topotecan alone but was no better than vincristine alone, which had progression of disease in only 1/7 mice tested (40).

### RH14

The RH14 xenograft was derived from an inguinal tumor in a patient previously treated with vincristine, dactinomycin, and cyclophosphamide, with complete response and no evidence of disease for 2.5 years, then recurrence. It had an embryonal histology based on histologic appearance of the original tumor and the xenograft, both of which showed differentiation and cross striations (36). In evaluations of tumor size injections of various chemotherapy agents, RH14 had complete regression with the use of vincristine and significant regression after cis-DDP, DTIC, and mitomycin-C. No growth inhibition was seen with dactinomycin or doxorubicin (32). It is important to note that RH14 was only established as a xenograft, and to our knowledge no cell line is available.

### RH36 (also referred to as Birch)

RH36 has an embryonal histology (based on the histologic appearance of the original tumor) and was derived from a paratesticular relapse in a 15-year-old male (41). The PPTP evaluated RH36 and found tumor xenograft inhibition in response to cyclophosphamide (41). RH36 has also been referred to as Birch in the literature (42) and is grown in RPMI-1640 with 10–20% FBS (43).

### RMS559/R31

RMS559 cells were established in the laboratory of Dr. Jonathan A. Fletcher, Department of Pathology, Brigham and Women’s Hospital, Boston, MA, USA (44, 45). They can be grown in DMEM with 10% FBS. This cell line was identified to have a V550L FGFR4 mutation by direct sequencing (46).

### RMS-YM

RMS-YM was derived from a relapsed abdominal mass of 2-year-old Japanese boy, at second relapse (47). The tumor was believed to originate from the urachus and was given an embryonal classification based on histology. The patient was treated with chemotherapy and radiotherapy, but died 3 years later due to multi-organ metastasis. RMS-YM is capable of forming subcutaneous xenografts in nude mice that appear histologically similar to the primary tumor. These cells stain positive for desmin and myoglobin. The karyotype of RMS-YM is 55, XY, −1, −2, −7, +8, −9, −10, −11, −16, −20, −22, +17 mars. Analysis of the *MYCN* and *RAS* genes showed no amplification or rearrangement, nor mutations of the 12th, 13th, or 61st codons of *KRAS*, *HRAS*, or *NRAS*. *TP53* pathway gene analysis showed no mutation of the *TP53* gene, but did show upregulation of *MDM2* (31). RMS-YM also shows overexpression of *FGFR1*, located in 8p11.2 and is grown in RPMI-1640 with 10% FBS (28).

### RUCH2

RUCH2 was derived from a 15-month-old female with a botryoid RMS of the vagina (48). It was originally established as a xenograft, then grown in cell culture. Karyotype at passage three revealed 55, X, –X, +7, +inv(7)(q11q32), +8, +8, +12, +13, +13, +19, +20, +21/55, idem, del(20)(q13) [cp40], almost identical to the primary tumor. It can be grown in DMEM supplemented with 10% FBS.

### RUCH3

RUCH3 was derived from a patient with relapsed embryonal RMS. This cell line is first mentioned in the original reference for RUCH-2 cell line, and was found to express PAX3 but not MYF3 or MYF5 (48). It can be grown in DMEM supplemented with 10% FBS.

### SCMC-RM2 (and subclone SCMC-RM2-1)

SCMC-RM2 was derived from the bone marrow of an 11-year-old female with relapsed, metastatic embryonal RMS. This patient had a primary tumor in her right abdominal wall with extensive marrow involvement. The tumor was classified as embryonal based on the presence of cytoplasmic longitudinal fibers and cross striations, as well as cell appearance using electron microscopy. She achieved remission with standard chemotherapy but relapsed and died 11 months later after diagnosis. Tumor cells from her bone marrow at relapse were obtained and cultured as SCMC-RM2. Subsequently, at passage 20, a parent cell line was cloned in soft agar and SCMC-RM2-1 was isolated. Both cell lines stain positive for muscle markers including desmin, vimentin, myoglobin, and actin. Interestingly, molecular and cytogenetic evaluation revealed amplification of the *MYCN* gene with overexpression of *MYCN* mRNA, which was also found in the primary tumor (49). SCMC-RM2 and its subclone can be grown in RPMI-1640 with 10% FBS (50).

### SMS-CTR

SMS-CTR was derived from an untreated 1-year-old male with a pelvic mass near the prostate. The original biopsy specimen is histologically consistent with a poorly differentiated embryonal RMS. This cell line was established by Dr. Patrick Reynolds in 1979. Cytogenetics were performed on this line in the 10th passage and showed hypertriploid chromosomes, with translocations involving t(1;13)(q21;q14) and t(2;13)(p25;q14) (51). It is important to point out that these are not the characteristic translocations associated with alveolar RMS. Though FOXO1 does localize to the 13q14 region, Northern blot studies of this line with a FOXO1 probe failed to show any evidence of a novel-sized transcript, thus suggesting that FOXO1 is not affected by these translocations (52).

### TTC442

TTC442, also occasionally called A91 in the literature, has an embryonal histology and is grown in RPMI-1640 with 10–20% FBS. This cell line was originally obtained from the laboratory of Dr. Tim Triche of Children’s Hospital Los Angeles (CHLA) (43, 53).

### TTC516

TTC516, also known as A995 in the literature, has an embryonal histology and is grown in RPMI-1640 with 10–20% FBS. This cell line was originally obtained from the laboratory of Dr. Tim Triche of CHLA (43, 53).

## Alveolar RMS Cell Lines

### CW9019

CW9019 has an alveolar histology and was developed by Dr. Jaclyn Biegel at the University of Pennsylvania, Philadelphia, PA, USA. It can now be obtained from Dr. Frederic Barr at the National Cancer Institute, Bethesda, MD, USA (personal communication, Dr. Frederic Barr). CW9019 has a stable reciprocal translocation of t(1;13), expresses the resulting PAX7-FOXO1 fusion protein and is grown in DMEM with 10% FBS (28). Unlike the typical PAX7-FOXO1-containing tumor, this line does not show amplification of the *PAX7-FOXO1* fusion gene (54).

### D-RHA1

D-RHA1 has an alveolar histology and was originally described in (43), having been obtained from Dr. W. Benedict of M.D. Anderson Cancer Center. It is grown in RPMI-1640 with 10–20% FBS.

### KFR

KFR was derived from a bone marrow metastasis of a 13-year-old female and designated RMS based on positive staining for vimentin and desmin. Alveolar histology was assigned based on morphology and upon identification of the t(2;13)(q37;q14) translocation, expressing the resulting PAX3-FOXO1 (formerly known as PAX3-FKHR) fusion protein (55). Although there was a time that the 2q breakpoint was assigned to q35 or q37, this has now been resolved as a q35 breakpoint, so that the translocation would now more correctly be stated as t(2;13)(q35;q14). KFR is grown in modified DMEM with 10% FBS (22).

### RH5

RH5 has an alveolar histology and has been cited in a study of the role of PDGFR-A (56).

### RH10

RH10 was derived from a 15-year-old female with a perineal tumor at relapse; she had previously received extensive chemotherapy with vincristine, cyclophosphamide, dactinomycin, and doxorubicin. RH10 was first established as a xenograft. The resulting cell line is hyperdiploid and cytogenetic analysis revealed t(2;13)(q35;q14) (57). Although the tumor from which RH10 was originally derived was described as having poorly differentiated embryonal histology, in another publication from the SJCRH group at the same time, this tumor was diagnosed as mixed alveolar/embryonal or monomorphous round cell. With the presence of the t(2;13) translocation, it should not be called embryonal RMS, but either mixed or alveolar RMS (39). After injections of various chemotherapy agents, complete tumor regression was seen after administration of L-PAM, but only transient growth inhibition, if any, to the other agents tested (58). RH10 has also been evaluated by the PPTP and found to have intermediate response activity to vincristine and high response to cyclophosphamide (41).

### RH18 (fusion-negative and fusion-positive variants)

The RH18 cell line was generated from a xenograft derived from a tumor from a previously untreated 2-year-old patient with a perineal RMS assigned as embryonal histology. Interestingly, there was a small amount of alveolar histology in the tumor (although the xenograft from which the cell line is derived was entirely embryonal) (36). At the time, such a mixed presentation often received a final diagnosis of embryonal RMS, though today the mixed tumors are considered an intermediate subset in which a fraction have the *PAX-FOXO1* fusion.

RH18 cell-line chromosome number ranges from 70 to 80 per cell, and cytogenetic abnormalities include del(1)(p22), der(3)t(3;?)(p14;?), del(6)(q24), and der(22)t(1;22)(q31;q11) (57). In the original cytogenetic characterization of this cell line, a t(2;13) translocation was identified in a later passage of a melphalan-resistant population (39). This sub-line also had cytogenetic markers in common with an earlier passage, suggesting that a translocation-bearing clone was selected (or arose) during culture. This finding is consistent with the presence of two available cell lines called RH18 – one fusion-negative (59) and one fusion-positive (60). Indeed an experiment comparing the parent xenograft to tumor selected for resistance after a large single dose of vincristine did show near tetraploidy (with numerous structural abnormalities) in the parent tumor, with all normal diploid cells noted in the resistant xenograft (61). This supports the idea that the original tumor, from which the xenograft was directly derived, had multiple stem lines.

The RH18 cell line also harbors amplification of the 12q13–15 region (including MDM2) (62), although unpublished data shows that the amplicon is present in the fusion-negative, but not fusion-positive, version of RH18 (personal communication, Dr. Frederic Barr). Clearly, Short Tandem Repeat (STR) analysis (see Section “Potential Pitfalls”) of the RH18 variants would be helpful to prove their relationship to the original xenograft, and users of these cell lines are well-served to determine which variant they are employing in their experiments.

When tumor volumes were measured following injections of various chemotherapy agents, RH18 showed growth inhibition with vincristine, cyclophosphamide, dactinomycin, doxorubicin, L-PAM, DTIC, cis-DDP, and mitomycin-C. No tumor growth inhibition was seen with bleomycin or busulfan (32). When evaluated by the PPTP, RH18 showed intermediate response activity to vincristine and high response to cyclophosphamide (41).

### RH28 (derived from the same patient as RH3)

The RH28 cell line was derived from a xenograft of tumor tissue obtained from an axillary node metastasis in a previously untreated 17-year-old male. The tumor and subsequent cell line have a poorly differentiated alveolar RMS histology. RH28 cells are near tetraploid and harbor a t(2;13)(q35;q14) translocation (57). Evaluation of RH28 proliferation *in vitro* shows that between passages 59–63, doubling time progressively increases until growth stops completely, and the cells appear to senesce. Further, these cells are multinucleated and show morphologies comparable to normal myoblasts (63). RH28 had complete volume regression with vincristine and L-PAM and significant growth inhibition with cyclophosphamide, doxorubicin, and dactinomycin (58). When tested by the PPTP, RH28 showed high response to both vincristine and cyclophosphamide (41).

There exists a sub-line of RH28, denoted RH28/LPAM, which was generated from RH28 cells in xenografts exposed to increasing doses of melphalan. RH28/LPAM cells are available for use and do not differentiate or stop proliferating with time (61). There are also available cells designated RH3, which have the same DNA breakpoint and pattern of DNA polymorphisms as RH28 cells (64). Indeed the RH3 cell line was developed by the Douglass laboratory from the same patient from which the RH28 xenograft was developed. The RH3 cell line has no relation to the Rh3 xenograft described in (36).

### RH30 (derived from the same patient as RMS13) and RH30R

The original Rh30 xenograft was established from an overnight culture of bone marrow obtained from a 16-year-old male with untreated metastatic alveolar RMS. The xenograft tumor was wild-type for *TP53*. The RH30 cell line was derived from the same patient bone marrow sample maintained in culture in the Douglass laboratory. The RH30 cell line expresses the t(2;13) translocation (39) and chromosomal analysis showed near-triploid chromosomes, between 51 and 87 (65). This line also has amplification of the 12q13–q15 region involving CDK4 and surrounding loci (66–68), and a heterozygous mutation of the *TP53* tumor suppressor (30, 31, 65). An independent cell line, RH30R, was derived from a xenograft, made from this same patient, at the time of relapse (41). The relapse xenograft and the cell line from the xenograft both have the same *TP53* mutation as the original RH30 cell line. RH30 and RH30R are cultured in RPMI-1640 with 10% FBS.

When the RH30 xenograft was tested with various chemotherapy agents, it showed complete regression using vincristine and L-PAM, significant growth inhibition after cyclophosphamide, but no growth inhibition with dactinomycin or doxorubicin (58). The PPTP evaluated the RH30 cell line and found it to have high response activity to vincristine and cyclophosphamide (41). A third experiment used the RH30 cell line to test the combination of topotecan plus vincristine against tumor growth. The combination showed better tumor control than topotecan alone but was no better than vincristine alone, where 0/5 mice tested had tumor progression (40). The RH30 cell line was also used to investigate the anti-tumor effects of tolfenamic acid. After treatment, RH30 cells showed decreased growth, decreased cell migration, and decreased expression of the PAX3-FOXO1 fusion protein (33).

An alveolar RMS cell-line labeled RMS13 (39, 69) is thought by some investigators to be related to RH30, perhaps derived from the same patient tumor as RH30, but this is not clear. Other studies have presumed RMS13 and RH30 to be independent entities (70). Finally, another cell line has been distributed with the name of RH30 (personal communication, Dr. Frederic Barr). Of interest, this other line still has the 12q amplicon but does not have the PAX3-FOXO1 fusion (unpublished data, Dr. Frederic Barr). STR analysis would help clarify the identity of both the RMS13 and fusion-negative RH30 lines.

### RH41 (derived from the same patient as RH4)

RH41 was established by the Houghton laboratory from a xenograft of a lung metastasis from a previously treated 7-year-old female patient with alveolar RMS. RH41 was originally designated as alveolar by histology, and more recently has been shown to harbor the t(2;13) translocation (71). RH41 also harbors mutation of the *TP53* gene (26). RH41 was tested by the PPTP and showed intermediate response activity to both vincristine and cyclophosphamide (41).

The cell line designated RH4 was developed from the same patient by another laboratory at SJCRH (personal communication, Dr. Peter Houghton), in which the tumor specimen was placed directly into tissue culture. These cells designated RH4 possess the t(2;13)(q35;q14) translocation (72) and have amplified, overexpression of EYA2 (28). RH4 cells are cultured in RPMI supplemented with 10% FBS.

### RH65

RH65 was derived from 18-year-old female with a relapsed RMS; it has an alveolar histology based on the original tumor (41).

### RMZ-RC2

RMZ-RC2 was established as a subclone of the RMZ cell line, which was derived from a bone marrow metastasis of a 2-year-old male with alveolar RMS; the child had been treated prior with chemotherapy and radiation (73). RMZ-RC2 can be grown in DMEM with 10% FBS. Chromosomal analysis showed near tetraploid range chromosomes with numerous monosomies and double minutes (65). Both the *PAX7-FOXO1* fusion gene and *MYCN* were found amplified (65, 72). RMZ-RC2 cells treated in culture with 2–5 Gy of radiation show dose-sensitive increases in the expression of myosin and the percentage of multinucleated cells, both markers of differentiation (74).

### TC212

TC212 has an alveolar histology (based on histology of the tumor specimens) and was derived from the bone marrow metastasis of a 16-year-old male who relapsed 5 months after multi-agent chemotherapy and radiation (51). Chromosomal analysis revealed multiple translocations, inversions, and deletions including what was thought to be t(2;13)(q37;q14). Although the 2q breakpoint was assigned to q35 or q37, this has now been resolved as a q35 breakpoint, so the translocation in TC212 should correctly be described as t(2;13)(q35;q14). TC212 cells are grown in RPMI-1640 supplemented with 10% FBS.

## RMS Cell Line Imposters

### A204

A204 was previously (but no longer) listed in the ATCC catalog as an RMS cell line. However in 1998, A204 cells were found to lack expression of myogenin, MyoD, or desmin, suggesting that either they were not of RMS origin or that with passaging in culture there was down-regulation of myogenic genes (23). These observations were especially compelling, since a prior immunohistochemical study found that most RMS tumor samples (but not normal adult muscle including skeletal muscle) expressed MyoD (75). In 2002, A204 cells were found to have a *SMARCB1* (SNF5) mutation, consistent with its classification as a rhabdoid line (76).

### A673

A673 was also previously (but no longer) listed in the ATCC catalog as an RMS cell line. However, like A204 cells, in 1998 these cells were found to not express myogenic markers (23). In 2003, A673 cells were found to express the *EWS-FLI1* fusion gene, indicating the cell line is derived from a Ewing sarcoma (77).

### RH1

The RH1 cell line was derived from a patient tumor specimen grown directly into tissue culture. The tumor was of the hand, described in a pathology report to be consistent with alveolar RMS by histologic criteria (personal communication, Dr. Peter Houghton). RH1 has no relation to the Rh1 xenograft described in (36). In 1998, RH1 was noted to lack expression of the myogenic markers myogenin, MyoD, and desmin (23). In 2008, using gene expression profiling the PPTP determined RH1 to cluster with tumors in the Ewing sarcoma family (4). RH1 was later verified to express the *EWS-FLI1* fusion characteristic of the Ewing family of tumors (78). In retrospect, it is not clear whether RH1 was contaminated by a Ewing cell line or mislabeled, however the PPTP has excluded RH1 from future RMS studies (4).

### Culture of RMS cell lines as rhabdospheres versus adherent cells

While all above mentioned studies propagated RMS cell lines as adherent cells or xenografts, Walter et al. recently described a method of culturing rhabdospheres in suspension (79). In this method, spheres are grown by placing human embryonal RMS cell lines into SC serum free medium with neurobasal medium and 10 ng/ml EGF, 20 ng/ml b-FGF, and 2× B27. In this study, RD cells were injected as adherent cells or spheres into mice, whereby tumor growth was measured over weeks. Sphere-derived tumors grew sooner, 40 days after injection, as opposed to 80 days after injection with the adherent cells. Sphere-derived tumors also required fewer cells for injection than adherent cells, indicating that rhabdospheres are more tumorigenic than adherent cell cultures. When stem cell genes associated with the stem cell phenotype were assessed using real-time PCR, sphere cultures had significantly higher expression of these genes than adherent cells, consistent with the premise of stem cell enrichment. Further, when treated with retinoic acid or DMSO, sphere cultures showed greater differentiation toward myocytes, neuronal cells or adipocytes, again indicating higher cancer stem cell potential in lines grown as rhabdospheres versus adherent cells. These data are compelling and may lead to changes in how RMS cell lines are grown for use in preclinical experimentation.

### Potential pitfalls

Several potential problems arise when using cell lines derived from human tumors *in vitro* or transplanted into mice as xenografts. One possibility is that tumors can be contaminated by murine stromal cells, thereby potentially affecting the outcome of the study. To combat this problem, analysis for species-specific LDH isoenzymes, as well as evaluating for acrocentric mouse chromosomes, can be performed. For example, Hazelton et al. cultured rhabdomyoblasts from the cell lines RH10, RH18, and RH28 and found human specific isoenzymes of LDH present in the lines, without evidence of murine LDH. In this same study, metaphase spreads were analyzed for acrocentric mouse chromosomes, and none of the three cell lines evaluated showed murine cells (57).

A second problem is that there may be discordance between the original tumor and the resulting cell lines or xenografts. To evaluate for this, one can perform gene expression microarray analysis. In 2008, the PPTP published a report using microarray analysis to analyze gene expression of the RMS cell lines RH10, RH18, RH28, RH30, RH41, RH65, and RD and compared expression profiles to a similar number of RMS specimens representing both alveolar and embryonal RMS. After exclusion of immunosurveillance genes (such as macrophage or B-cell markers), the cell lines resembled the other human RMS specimens (4). A similar analysis was performed using cDNA microarray analysis of the RMS cell lines RH1, RH6, RH10, RH12, RH14, RH18, RH28, RH30, RH36, and RH41. These analyses were compared to cDNA of primary tumors and all clustered appropriately by tumor type except RH1 and RH6 (37). Further cDNA microarray analysis done by Khan et al. showed comparisons between alveolar cell lines RMS13, RH3, RH4, RH5, RH18, and RH28 (69). Missiaglia et al. performed cDNA microarray analysis and gene expression profiles, and compared these profiles to primary tumors on the RMS cell lines RD, RMS-YM, RUCH2, RUCH3, RH4, RH18, RH30, RH41, and CW9019 and showed high concordance as well (28). A recent study describes an approach to develop xenografts directly from patient tumors, then test them for genomic similarities (80). In this study, after surgical resection from human subjects, tumors were either implanted into immune-compromised mice as xenografts or analyzed for histology, mutational analysis and gene expression profiles. Xenografts were later surgically resected and implanted into a second set of mice. Xenograft specimens from first and second generation implantations underwent similar analysis as the original patient tumor sample. This approach serves as a good model for evaluating and validating human tumor xenografts, which may help produce preclinical data more closely mirroring later clinical outcomes

A third problem is the situation in which two cell lines with different names are apparently from the same tumor, such as RH36 and Birch, RH30 and RMS13. This situation can represent two different nomenclatures or possibly contamination of one cell line with the other. In addition, although RH3 and RH28 appear genetically identical by fusion breakpoint and STR analysis (64, 81), it is not clear whether the two cell lines were derived from the same tumor from the same patient on the same date. In either case, the two lines may share much of their genetics but may also have diverged biologically due to continued growth as separate cultures. There are also instances where two distinct lines have the same name, but have different genetics. For example, the “RH18” cell line can refer to the version not bearing the fusion gene (59), or the version bearing the fusion gene (60), which may have emerged from the original cell culture.

A fourth problem is unintentional cross-contamination between cell lines. It is estimated that 15–35% of all cell lines are of a different parental origin than assumed, and this results in the misidentification of cell lines and ultimately the misinterpretation of scientific data (82–84). For example, the cell line TE671, which was widely used to study medulloblastoma, was thought to be a misidentified RMS cell line (RD). This statement stemmed from similar phenotypic observations and an activating point mutation in NRAS gene which was found both in TE671 and RD (34). This hypothesis was confirmed with a cytogenetic study of these cell lines (85). In addition, three esophageal adenocarcinoma cell lines were recently found to derive from other tumors (86).

Because of the problems described above, and no historical standard method to validate cell lines, the method of STR profiling was suggested as an international reference standard for cell line authentication (87). STRs (or microsatellite repeats in DNA) consist of 2–6 base pair repeats that differ from individual to individual (84). In this method, commercially available primers are used to amplify a number of polymorphic STR loci, resulting in a cell-line specific “fingerprint” that is recorded as a numerical code in a reference database. The simplicity of the code, reproducibility, and low cost of this method are the strengths supporting its use as a standard of cell line authentication (87). Indeed, several scientific journals including those managed by the American Association for Cancer Research are requiring that articles submitted for peer review contain documentation of cell line authentication (see http://www.aacrjournals.org/site/AuthServCtr/cell_line_auth.xhtml). On the other hand, it is important to acknowledge the limitations of STR in cell line authentication. For example, in a study of four human leukemia cell lines grown under variable culture conditions, STR profiles changed in two of the four cell lines due to subclone outgrowth (84). Thus, STR profiling is an important tool for cell-line validation, but has limitations, especially in tumor cell lines subject to experimental conditions that could alter cellular genetics. Other features including myogenic markers, enzyme isotypes, and cytogenetic analysis can further assist in validation of RMS cells when differences are found in STR loci (57, 84). Table 3 summarizes the validation methods that have been employed for human RMS cell lines. The cell lines with known and published STRs are listed in Table 4.

**Table 3:** **RMS cell lines: validation methods performed to date**.

Cell lines	LDH isoenzymes	Microarray analysis	Short tandem repeat
**EMBRYONAL HISTOLOGY**			
CCA	–	–	–
CT-TC	–	–	–
HX170c	–	–	–
JR-1	–	–	–
KF-RMS-1	–	–	–
RD	–	Yes	Yes
RH6	–	–	–
RH12	–	Yes	–
RH14	–	Yes	–
RH36 (Birch)	–	Yes	–
RMS559/R31	–	–	–
RMS-YM	–	Yes	–
RUCH2	–	Yes	–
RUCH3	–	Yes	–
SCMC-RM2	–	–	–
SMS-CTR	–	–	Yes
TTC442	–	–	–
TTC516	–	–	–
**ALVEOLAR HISTOLOGY**			
CW9019	–	Yes	–
D-RHA1	–	–	–
KFR	–	–	–
RH5	–	Yes	–
RH10	Yes	Yes	–
RH18	Yes	Yes	Yes
RH28 (RH3)	Yes	Yes	Yes
RH30 (RMS13)	–	Yes	Yes
RH41 (RH4)	–	Yes	Yes
RH65	–	Yes	–
RMS13	–	Yes	–
RMZ-RC2	–	–	–
TC212	–	–	–

**Table 4:** **Available RMS cell line short tandem repeats**.

Cell line	D8S1179	D21S11	D7S820	CSF1PO	D3S1358	TH01	D13S317	D16S539	D2S1338	D19S433	vWA	TPOX
RH18	13, 15	32.2, 33.2	8, 10	10	16	7	12	9, 12	19, 24	12, 14	15, 17	8, 9
SMS-CTR	10, 12	29, 31.2	8, 11	12	17	6	11	10, 11	24	14	18, 19	8, 12
RH28 (RH3)	12, 13	31.2, 32.2	10, 11	10, 12	14, 17	7	12, 13	11, 12	18, 25	14, 15.2	14, 17	8, 9
RH30 (RMS13)	12, 15	29, 31.2	10	10, 11	15	9, 9.3	11	12	17, 20	14, 15.2	17, 18	8, 11
RH41 (RH4)	10, 13	29, 31	10, 11	11, 12	17	7, 9.3	8, 9	12, 13	17, 21	13, 15	16, 18	8, 11
RD	11, 15	28, 29	8, 12	10, 11	15, 17	9.3	13	10, 11	17, 23	11, 14	18	9

[(35); www.COGcell.org].

## Discussion

Numerous RMS cell lines are available for use in basic research and preclinical testing. These cell lines differ markedly in their histologies, origins, karyotypes, degree of treatment prior to development, and the levels to which these lines have each been validated and compared to their tumors of origin. This information is of critical importance when choosing a cell line for study. For example, evaluation of results of studies performed to date on the available cell lines shows a trend that lines derived from tumors previously treated with extensive chemotherapy and/or radiation are then less susceptible (or only transiently so) to standard agents. These previously treated cell lines would therefore be optimal for preclinical testing of new agents proposed for use in relapsed or refractory disease. These further progressed tumors may also be easier to establish in culture than primary tumors. It is necessary to ensure that cell lines used in preclinical trials closely resemble the tumors from which they were derived. While some data is currently available that validates several lines, this work is ongoing and will surely continue to develop as the use of cell lines and RMS xenografts increase in pediatric oncology research.

When examining RMS cell lines as a whole, there is a surprising lack of cell lines derived from primary, untreated RMS tumors. Indeed, the majority of both embryonal and alveolar RMS cell lines are established from tumor relapses or distant metastases. Cells that arise from relapses or metastases (in addition to any adaptations as the result of radiation and/or chemotherapy given to the patient) have genetic changes distinct from the primary tumor, and may be easier to establish in culture than cells from primary tumors. Therefore, applicability of these lines in preclinical studies examining RMS primary tumor biology is questionable. This gap in our understanding of RMS reveals a significant challenge to researchers that routinely use RMS cell lines for preclinical research. How can we make significant strides to identify therapeutic targets when the reagents available do not accurately represent the biology of the primary tumor? Has RMS research been hindered by an excess of false negative hits in pharmacologic and genetic screens due to these cell-line adaptations?

In summary, it will be important for RMS scientists, as a community, to critically evaluate the cell lines we use, and identify approaches to generate improved reagents for future research. Ideally, human cell lines would be established from primary, untreated RMS tumor biopsies. Then, any cell lines generated from subsequent tumor relapse or metastasis could be paired with the primary cell line as a “matched set.” To our knowledge there are no matched set cell lines publicly available to RMS researchers. When available, such a resource would support the study of the genetic and biologic changes in the primary tumor, as well as the changes leading to recurrence or metastasis.

## Conflict of Interest Statement

The authors declare that the research was conducted in the absence of any commercial or financial relationships that could be construed as a potential conflict of interest.
